# Cognitive Functioning in Abstinent Patients with Alcohol Use Disorder: Exploring Evidence for Premature Aging

**DOI:** 10.3390/brainsci16030320

**Published:** 2026-03-17

**Authors:** Jeroen Staudt, Yvonne C. M. Rensen, Hein A. De Haan, Jos I. M. Egger, Boukje A. G. Dijkstra

**Affiliations:** 1 Tactus Institute for Addiction Care, 7418 ET Deventer, The Netherlands; jeroen.staudt@donders.ru.nl (J.S.); hadehaan56@gmail.com (H.A.D.H.); 2 Donders Institute for Brain, Cognition and Behaviour, Radboud University, 6500 HD Nijmegen, The Netherlands; yvonnerensen@vigogroep.nl (Y.C.M.R.); jos.egger@donders.ru.nl (J.I.M.E.); 3 Centre of Excellence for Korsakoff and Alcohol Related Cognitive Disorders, Vincent van Gogh Institute for Psychiatry, 5803 AC Venray, The Netherlands; 4 Forensic Psychiatry Department de Boog, GGNet, 7231 PA Warnsveld, The Netherlands; 5 Centre of Excellence for Neuropsychiatry, Vincent van Gogh Institute for Psychiatry, 5803 DN Venray, The Netherlands; 6 Nijmegen Institute for Scientist Practitioners in Addiction, Radboud University, 6503 GK Nijmegen, The Netherlands; 7 Novadic-Kentron, Addiction Care Center, 5261 LX Vught, The Netherlands

**Keywords:** alcohol use disorder, aging, cognitive, contextual neuropsychology

## Abstract

**Highlights:**

**What are the main findings?**

- Cognitive aging in abstinent patients with AUD may be domain-specific.
- Perceptual reasoning and recovery may be affected by increasing age.

**What are the implications of the main findings?**

- The findings are not considered strong enough to evidence the vulnerability hypothesis and do not support accelerated aging at all.
- Replication of the findings is needed with larger sample size and the inclusion of older adults (>60 year).

**Abstract:**

Background/Objectives: Chronic alcohol use accelerates biological and cognitive aging, yet it remains unclear how cognitive aging progresses during abstinence in alcohol use disorder (AUD). It is also unknown to what extent this follows models such as accelerated aging or the age-related decline as proposed by the vulnerability hypothesis. This study examined age-related changes and cognitive recovery during abstinence in patients with AUD. Methods: A total of 197 clinically admitted patients, referred for detoxification and extensive neuropsychological examination, were included. Neuropsychological testing was administered in the second and sixth week of admission using well-normed instruments. Using both multi-assessment and cross-sectional data, relationships between age and normed cognitive outcome scores were examined. Results: After six weeks of abstinence, age-related deviations were observed for perceptual reasoning (PRI), verbal comprehension (VCI), and short-term memory (SMI) but not for ten other cognitive indices. During admission, age significantly influenced the change in belonging to a specific recovery category. Each additional year of age reduced the odds of showing no cognitive impairment by 5% and reduced the odds of cognitive recovery by approximately 4%, compared to non-improvers. Conclusions: Age-related influences appear limited to specific cognitive functions and do not follow a uniform or easily interpretable pattern. Perceptual reasoning seems negatively affected after age 60 for participants with six weeks of abstinence. Older participants showed a reduced likelihood of cognitive recovery and a reduced likelihood of having no cognitive problems at all. The findings do not support accelerated aging and are still too weak to be considered evidence for the vulnerability hypothesis. Implications for future research are discussed.

## 1. Introduction

Normal, healthy aging is associated with neurobiological alterations, decline in various cognitive functions, and a susceptibility to develop neurodegenerative disorders such as Alzheimer’s disease [1,2,3,4]. However, there is considerable heterogeneity among older adults, and a substantial proportion maintains well-preserved cognitive functioning without evidence of impairment. Healthy lifestyle factors including, e.g., physical fitness, may contribute to retaining or even regaining cognitive abilities. Conversely, evidence suggests that chronic and excessive alcohol use can worsen cognitive processes, leading to accelerated biological aging [5,6,7] and a faster decline in cognitive functioning with age [3,8,9]. The relationship between aging and cognitive functioning in patients with alcohol use disorder (AUD) has been examined through the ‘premature aging’ hypothesis, which proposes that cognitive functioning of patients with AUD resembles that of older adults without AUD [10]. Findings from older studies investigating this hypothesis are mixed, ranging from full support [10], to partial [11], and no support [12,13].

The premature aging hypothesis has been refined into an *accelerated aging hypothesis* and a *vulnerability hypothesis* [14,15,16]. The accelerated aging hypothesis proposes that cognitive performance of the individual with AUD resembles that of an older normal person from the onset of chronic and excessive drinking, irrespective of a specific age. Three studies were found with evidence that may be considered to support accelerated aging in individuals with excessive alcohol use. A recent, longitudinal cohort study, measuring global intelligence during early adulthood and during late midlife, showed increased impairments of global cognitive functioning (0.12 point decline in IQ score) in men with heavy alcohol use (about 40 g per day) compared to men with lower weekly alcohol consumptions (1–14 units/week) [17]. In another 10-year longitudinal cohort study, participants with heavy alcohol use (≥36 g per day) showed, with increasing age, a faster decline in global cognitive functioning (2.4 extra years), short-term verbal memory (5.7 extra years) and executive functioning (1.5 extra years), compared to participants from less severe categories [18]. These findings illustrate a relation between the severity of alcohol use and cognitive performance, which translates into performance consistent with an older age. An older cross-sectional study reported that, regardless of their age, patients with AUD performed worse on a series of neuropsychological tasks than their age-matched controls, supporting accelerated aging [19]. The number of studies supporting accelerated aging is small, and they investigated only a narrow range of cognitive domains. For example, visuospatial functions and auditory or visual memory functions were either not examined or only minimally addressed, whereas these functions are considered characteristic cognitive domains impaired in patients with AUD [9].

The vulnerability hypothesis proposes that chronic and excessive drinking at an older age leads to substantially greater impairments compared to individuals at a younger age. Also here, only three studies have investigated the implications of this hypothesis, with mixed findings [19,20,21]. While results from the Noonberg et al. [19] study did not support the hypothesis, Kurihara et al. [20], using a cognitive screening instrument for patients with schizophrenia, found a tipping point in which participants of 53 years and older showed greater cognitive impairments (>2 SD) from age-related norms compared to younger participants (<2 SD). The contradictive findings may be partially explained by the limited range of cognitive domains assessed, the use of less suitable instruments to tap cognitive domains, or the absence of control groups. Another factor influencing the findings may be the period of abstinence, with considerable variation in abstinence duration [19] or the relatively short abstinence period of 4 weeks [20]. This may have influenced cognitive performance due to neurobiological disruptions occurring during detoxification [22]. In addition, cognitive impairments may (partly) resolve following prolonged abstinence, also influencing the relation between age and cognitive functioning [23]. From their longitudinal study examining abstinent older patients with AUD (mean age 71), Ross Cucurull et al. [21] tentatively suggested that recovery of cognitive functioning in older patients occurs more slowly, affecting the relation between age and cognitive performance. For this, however, they found no empirical support.

Despite long-standing interest in aging in patients with AUD, the studies discussed above do not provide information on two important points. Hallmark cognitive domains (e.g., memory, visuospatial functioning) were not or only marginally investigated, and duration of abstinence has not been sufficiently addressed. Cross-sectional studies employed short-term abstinence or considerable variation in abstinence duration, while the longitudinal studies did not investigate within-subject changes. While neurobiological studies support both accelerated aging and a vulnerability to brain aging with increasing age [5,24], it remains unclear how cognitive aging unfolds in abstinent patients with AUD, whether it follows an accelerated pattern or is characterized by significant deterioration at a particular age. The aim of this study is to examine age-related deviations during recovery of cognitive functions using a prospective cohort study in a defined period of abstinence. To this end, we investigate the relation between age and cognitive functioning ((working) memory, visuospatial functioning, speed, intelligence) after six weeks of abstinence and the relation between age and change in cognitive functioning during that same period. Identified relationships are controlled for factors known to be associated with both cognitive functioning and age [25,26].

## 2. Materials and Methods

### 2.1. Design and Participants

This prospective observational cohort study was conducted at specialized clinical addiction healthcare facilities in the Netherlands (Tactus Institute for Addiction Care). Data from routine care processes were collected between November 2017 and August 2024. Patients with AUD admitted for clinical detoxification and neuropsychological examination were included. Patients were excluded in case of pregnancy, severe somatic or neurological disorders (e.g., lupus, congenital epilepsy, acquired brain injury), acute psychiatric disorders (psychosis, suicidal ideation), or insufficient Dutch language skills. The research was performed in accordance with the Declaration of Helsinki and approved by Tactus Institutional Review Board, executed following institutional guidelines. All participants gave written informed consent.

### 2.2. Procedure

Patients were referred to the clinical facilities for inpatient alcohol detoxification and extensive neuropsychological assessment. At admission, demographic information was collected, as well as information on alcohol, polysubstance use (cocaine, cannabis, MDMA, amphetamines), and tobacco use. As part of routine care, patients completed a cognitive screening using the Montreal Cognitive Assessment (MoCA) twice [27]: in the second and sixth week of admission. At one point in time, during the sixth week, patients underwent extensive neuropsychological assessment, including the WAIS-IV and WMS-IV. The timing of these assessments aligns with prior research and established recommendations on abstinence duration to minimize detoxification related cognitive disturbances [22,28]. By means of twice-weekly urinalysis, abstinence from alcohol and other substances during admission was verified. All neuropsychological tests were performed by a trained psychologist according to appropriate user guidelines [29,30].

### 2.3. Measurements

Information on age, education level and gender were registered as well as quantities of previous alcohol use (e.g., ‘the number of years of daily alcohol use’, ‘the age of onset of daily alcohol use’), all via a clinical interview based on the Measurements in the Addictions for Triage and Evaluation [31]. The education level was classified according to the seven categories model of Verhage [32] and subsequently merged into three categories, ‘Low’ (levels 1 and 2), ‘Average’ (levels 3, 4 and 5) and ‘High’ (levels 6 and 7). Substance use disorders were classified following DSM-5 criteria (American Psychiatric Association, 2013). All diagnoses were established by a certified clinical neuropsychologist.

#### 2.3.1. Montreal Cognitive Assessment (MoCA)

The MoCA is a concise screening tool for measuring cognitive functioning using various domains of neuropsychological functioning (visuospatial skills, attention, language, memory, abstraction and orientation) [27]. The uncorrected total score (0–30) was used as an outcome measure for the purpose of this research. A score < 26 indicates a possible cognitive impairment. The instrument is available in parallel versions (7.1, 7.2 and 7.3, 8.1 and 8.2). The 7.1, or more recently the 8.1 version, was used during the first assessment, and the 7.2 or 8.2 version during the second assessment. The instrument is translated into Dutch [33], is well normed [34], is validated and shows good test–retest reliability [35].

#### 2.3.2. Wechsler Adult Intelligence Score Fourth Edition (WAIS-IV)

The WAIS-IV is an individually administered test used to assess the cognitive and intellectual abilities of a person aged 16 to 85 years [36]. The WAIS-IV is widely used in neuropsychological assessments [37] and has acknowledged value when assessing neuropsychological dysfunction [38] and the relationship between aging and cognition [39].

For the purpose of this research we used the original four-factor model (Verbal Comprehension Index (VCI), Perceptual Reasoning Index (PRI), Auditive Working Memory Index (AWMI), Processing Speed Index (PSI)) and three additional indexes from the alternative five-factor model (Fluid Intelligence (FI), Short-term Memory (SMI), Visual Information Processing (VIP)) based on the Cattell–Horn–Carroll theory of cognitive abilities [40]. The other two indexes (Crystallized Intelligence (CI), Processing Speed (PSI)) were not used since these are identical to the VCI and PSI from the original model. Both models are considered complementary for interpreting WAIS-IV results [30,41,42]. A total of 10 core subtests (Similarities [SI], Vocabulary [VC], Information [IN], Block Design [BD], Matrix Reasoning [MR], Visual Puzzles [VP], Digit Span [DS], Arithmetic [AR], Coding [CD], Symbol Search [SS]), and three supplemental subtests (Figure Weights [FW], Picture Completion [PC], Letter-Number Sequencing [LN]), were administered. With these subtests, all indexes of both the four- and five-factor model could be calculated. Raw scores are translated to age-related normed scores (M = 100, SD = 15) using the WAIS-IV manual. Scores ≤ 76 are considered deviating from the norm (≤1.5 SD).

Reliability scores for the Dutch WAIS-IV version are considered good (varying around or above 0.90, and above 0.80 for split-half reliability), as are its criterium and construct validity properties [29].

#### 2.3.3. Wechsler Memory Scale Fourth Edition (WMS-IV)

The WMS-IV is an individually administered test used to assess auditive and visual memory (immediate retrieval (I), delayed recall (II) and recognition) and visual working memory functions. It has versions for adults (16–69 years) and older adults (65–90 years). For the purpose of this research, the latter version was only administered when the participant was 70 years or older. The adult version consists of six subtests, covering Auditive Memory (AMI) (Logical Memory I/II, Verbal Paired Associates I/II), Visual Memory (VMI) (Designs I/II, Visual Reproduction I/II) and Visual Working Memory (VWMI) (Serial Addition, Symbol Span). For each participant, a score was also calculated reflecting the capacity for immediate memory (Immediate Memory Index, IMI) and delayed memory (Delayed Memory Index, DMI). All raw scores were translated to age-related scores (percentiles) using Q-interactive software (https://qiactive.com/choose-share/login/auth (accessed on 1 March 2026)). Scores ≤ 5th percentile are considered deviated from the norm (≤1.5 SD). The version for older adults does not contain the Designs task and the tasks underlying the WMI.

The WMS is well normed, and the reliability of the index scores is considered good (internal consistency between 0.87 and 0.97, interrater reliability between 0.77 and 1.00) [43]. Together with the WAIS-IV, the WMS-IV provides a comprehensive evaluation of one’s neurocognitive status [38].

### 2.4. Sample Size

We determined our sample size using G*power (version 3.1.9.6) [44] and calculated an approximation of the sample size for an F test (multiple linear regression). Based on previous studies [17,18], we expected a small to medium effect size (f2 = 0.10). Alpha was set at 0.05, and we accepted a power of 0.80. We estimated the maximum number of predictors to be used between one and four, depending on the specific model. For the analysis of both our cross-sectional and longitudinal data, a sample size between 85 and 125 participants was calculated in order to detect an effect using multiple linear regression.

### 2.5. Outcome Measures

Cognitive functioning as the dependent variable is reflected by the normed scores on the WAIS-IV (Verbal Comprehension, Perceptual Reasoning, Working Memory, Speed, Full Scale Intelligence Quotient, Fluent Intelligence, Short Term Memory, Visual Performance) and WMS (Auditive Memory, Visual Memory, Short Term Memory, Immediate Memory, Extended Memory).

One variable was created reflecting cognitive recovery, consisting of four categories: improvement (group A), no improvement (group B), deterioration (group C) or no cognitive impairments (D). Group assignment was based on a combination of the two MoCA scores (week 2 and week 6 of clinical admission). Participants were assigned to group A when they scored <26 at the first assessment and ≥26 in the second, to group B when the first and second score were <26, to group C when the first score was ≥26 and the second score was <26, and to group D when both scores were ≥26.

Variables that are both associated with cognitive functioning and age were considered confounders. Demographic (gender, age, education level) and drinking-related (severity of alcohol use disorder, use of other non-prescribed substances, polysubstance use (yes/no), tobacco use disorder (yes/no), years of daily alcohol use, age of onset of daily alcohol use, and neurological injury (yes/no)) variables were considered in this regard. For all confounder analyses we used Chi-square tests (Somers’ d for ordinal X binary analyses, Fisher’s Exact Test for binary X binary analyses), parametric or non-parametric tests if applicable. To detect candidate confounders, we used a more tolerant *p*-value (*p* < 0.10) to minimize type II error.

### 2.6. Statistical Analysis

Baseline characteristics were described using the appropriate measures for descriptive statistics. The percentage of participants scoring ≥ 1.5 SD below normal indicated possible cognitive impairment, in line with other studies in the field [35,45]. Normality was judged by visual inspection of the data. In case of doubt, skewness and kurtosis were calculated to Z-scores [46]. A score between −2 and +2 was accepted for normality [47]. In case of missing data, variables were listwise removed from the analysis. When participants completed the older version of the WMS-IV, the Visual Memory Index score was not available for analysis. Given the exploratory character of our research, and to find new possible leads that could improve our understanding of premature aging, we did not correct for multiple testing [48].

We took the following steps to examine age-related deviations in relation to cognitive functioning after six weeks of abstinence. First, variables that could serve as candidate confounders in the relation between age and cognitive functioning were identified. Stratification of data was considered when a confounder substantially influenced the cognitive outcome measures, and the relation between the confounder and age, or between the confounder and the outcome measure, could not be interpreted simply as (non-)linear. Next, we analyzed whether age predicted cognitive functioning by means of a linear relationship, using linear regression analyses. Since the scores on cognitive functioning are already corrected for age, a significant model presents a deviation from the age norm. For every significant regression model found (*p* < 0.05), we examined the effect of candidate confounders on this relationship by adding one candidate confounder to the model at a time. During this step, a model and the contribution of age, or the confounder, are considered significant when *p* < 0.05. Finally, non-linear associations were examined using spline regression. Three excess-age predictors (excess40, excess50, excess60) were created, reflecting the numbers of years a participant’s age exceeded 40, 50 or 60 years, respectively, and coded as zero when age was equal to or below the reference value. For each cognitive outcome, a model including all three predictors was estimated. Significant models (*p* < 0.05) were interpreted as evidence for non-linear age-related effects. Given the possible high correlation among predictors, individual coefficients were not interpreted independently; instead, the pattern of coefficients was examined to characterize changes in the trajectory of cognitive functioning across age range (e.g., increases, plateaus, or declines after ages 40, 50 or 60). The R^2^-values were not interpreted due to possible overfitting.

To analyze the relation between age and neurocognitive recovery, we used a multinomial logistic regression analysis. We examined whether age predicted assignment to either one of the four recovery categories, using group B as the reference category (*p* < 0.05). Nagelkerke R^2^ was used to determine the goodness of fit, using the following qualification criteria: poor (0–0.1), modest (0.1–0.3), moderate (0.3–0.5) and strong (>0.5) [49]. Statistical analysis was carried out using the Statistical Package for Social Sciences software, SPSS, IBM (version 29).

## 3. Results

### 3.1. Descriptives of Participants

A total of 197 patients with (mostly severe) AUD were included in this study (see Table 1). Participants were on average 43.9 years old, with the majority under 60 years old (88%). Substantial amounts of participants (16.9–37.1%) were impaired (≤1.5 SD) on tasks; see Appendix A. Participants with a high education level did so on three out of thirteen tasks (10.0–13.3%). Not all outcome measures were available for all included participants for two reasons. First, intelligence testing, particularly extensive intelligence testing using all WAIS-IV subtests, was not conducted if participants had completed a comparable assessment within two years prior to admission. Second, fewer data were available for the WMS because this instrument was gradually introduced across clinics during the eight-year data collection period. Third, a total of 110 participants completed the MoCA at both assessment points, with 154 participants at the first time point and 112 participants at the second. Data were incomplete for several reasons, for example, due to illness, premature discharge, or the MoCA was not completed in full. The missing data showed a random pattern. Analysis comparing participants that completed both MoCA assessments with participants that completed only one, or none of the assessments, yielded no differences with regard to demographic variables (see Table 1 for demographic factors).

The MoCA scores showed no cognitive impairments for 47 participants compared to 34 participants with cognitive impairments on both time measurements; an improvement was seen for 19 participants, while 10 participants showed deterioration of cognitive function (see Table 2).

### 3.2. Confounders Influencing the Relationship Between Age and Cognitive Functioning

Confounder analyses revealed that education level, age of onset of daily alcohol use, and polysubstance use may influence the relationship between age and cognitive functioning (see Appendix A).

#### 3.2.1. Education Level

The effect of age on education level was significant (F (2, 192) = 3.812, *p* = 0.024). Bonferroni-adjusted post hoc comparisons showed that the high education level group was about 7.8 years older than the low education level group (MD = 7.8, 95% CI [0.94, 14.63], *p* = 0.02), whereas no significant difference was found between these groups with the average education level group (*p* = 0.46, *p* = 0.17). Education level affected 13 out of 15 (90%) of the outcome measures (*p* < 0.05), except VWMI. Since the relation between education level and age is not linear, or non-linear, data was stratified by education level, yielding different mean scores per outcome. Substantial amounts of participants with a low (39.4–71.4%) and average education level (14.3–34.9%) were impaired (≤1.5 SD) on all tasks (see Table 3). The age distribution across education levels was largely comparable, with a slightly higher proportion of older participants in the high-education level (see Appendix A).

#### 3.2.2. Age of Onset of Daily Alcohol Use

Age of onset correlated with both age (r (171) = 0.534, *p* < 0.001) and 12 out of 15 outcome measures (*p* < 0.05), except VPI, PSI and VWMI. When age of onset was added as a confounder to the models that showed significant associations with age, it did not contribute significantly to any of the outcomes.

#### 3.2.3. Polysubstance Use

Participants with polysubstance use were significantly younger (M = 38.0, SD = 11.3) than participants with AUD only ((M = 48.3, SD = 12.1), t (181) = 5.837, *p* < 0.001). Polysubstance use correlated with 5 out of 15 outcome measures (*p* < 0.05). When polysubstance was added as a confounder to the models that showed significant associations with age, it did not contribute significantly to any of the outcomes.

#### 3.2.4. Linear Effect of Age on Cognitive Functioning After Six Weeks Abstinence

After stratification for education level, regression analyses using age as the predictor for cognitive functioning indicated two indices (VCI, SMI) that were significantly predicted by age, both in participants with an average education level and those with a high education level (see Table 4).

In participants with an average education level, the SMI score increased by 0.303 points per year of age (F (1, 59) = 4.883, *p* = 0.031, 95% CI [0.03–0.58], R^2^ = 0.076). In participants with a high education level, age predicted the VCI score with an increase of 0.405 points per year of age (F (1, 29) = 6.450, *p* = 0.017, 95% CI [0.08–0.73], R^2^ = 0.182).

**Table 3:** Overview of outcome measures stratified per education level.

	Low Level of Education				Average Level of Education				High Level of Education			
	N	Mean	Min–Max	<1.5 SD (%)	N	Mean	Min–Max	<1.5 SD (%)	N	Mean	Min–Max	<1.5 SD (%)
WAIS-IV												
VCI	33	78.1	60–103	39.4	84	89.2	64–129	14.3	31	107.1	77–127	0
PRI	33	77.1	60–104	51.5	83	85.2	66–123	26.5	31	100.9	72–125	6.5
AWMI	33	77.3	55–114	42.4	84	85.5	55–122	20.2	30	102.3	68–125	3.3
PSI	33	78.9	45–114	48.5	85	85.7	48–134	25.9	30	94.3	59–141	10.0
FSIQ	33	73.9	59–108	63.6	83	82.6	8–116	34.9	28	101.4	80–129	0
FI	21	72.6	54–108	71.4	61	82.6	56–102	31.1	15	103.5	75–127	13.3
SMI	20	76.1	45–105	50.0	61	86.5	45–122	21.3	15	102.3	80–125	0
VPI	21	82.8	56–102	42.9	61	84.4	62–127	29.5	14	101.0	78–125	0
WMS-IV												
AMI	16	17.3	0–69	43.8	58	30.1	0–91	27.6	19	51.8	7–145	0
VMI	16	17.4	0–94	43.8	58	33.2	0–97	19.0	19	51.2	3–119	5.3
VWMI	12	22.0	0–85	50.0	42	28.5	0–113	21.4	15	36.0	1–112	13.3
IMI	16	14.2	0–78	62.5	58	28.3	0–86	31.0	19	53.7	4–142	5.3
DMI	16	16.2	0–79	43.8	56	29.6	0–95	30.4	19	49.5	4–130	5.3

**Notes:** for the purpose of this study, the original seven categories of education level according to Verhage [32] were merged into three categories: Low (levels 1 till 3), Average (levels 4 and 5), High (levels 6 and 7). WAIS-IV normed scores are shown using T-scores (Mean = 100, SD = 15). WMS-IV normed scores are shown using percentiles. Scores < 1.5 Standard Deviation, ≈7% in a normal distribution, are scores ≤76 or ≤5th percentile. **Abbreviations:** WAIS-IV, Wechsler Adult Intelligence Scale, fourth edition; VCI, Verbal Comprehension Index; PRI, Perceptual Reasoning Index; AWMI, Auditive Working Memory Index; PSI, Processing Speed Index; FSIQ, Full Scale Intelligence Quotient; FI, Fluid Intelligence; SMI, Short-term Memory; VIP, Visual Information Processing; WMS-IV, Wechsler Memory Scale fourth edition; AMI, Auditive Memory Index; VMI, Visual Memory Index; VWMI, Visual Working Memory Index; IMI, Immediate Memory Index; DMI, Delayed Memory Index; SD, Standard Deviation.

**Table 4:** Regression analysis cognitive functioning stratified by education level.

	Low		Average		High	
Outcome	*p*	R^2^	*p*	R^2^	*p*	R^2^
WAIS-IV						
VCI	0.303	0.034	0.147	0.025	0.017 *	0.182
PRI	0.504	0.015	0.198	0.020	0.150	0.070
AWMI	0.723	0.004	0.677	0.002	0.586	0.011
PSI	0.197	0.053	0.191	0.020	0.209	0.056
FSIQ	0.271	0.039	0.927	0.000	0.155	0.076
FI	0.919	0.001	0.275	0.020	0.280	0.089
SMI	0.345	0.050	0.031 *	0.076	0.829	0.004
VPI	0.744	0.006	0.686	0.003	0.132	0.179
WMS-IV						
AMI	0.998	0.000	0.411	0.012	0.596	0.017
VMI	0.820	0.004	0.511	0.008	0.993	0.000
VWMI	0.764	0.009	0.066	0.082	0.095	0.199
IMI	0.808	0.004	0.276	0.004	0.609	0.016
DMI	0.983	0.000	0.702	0.003	0.997	0.000

**Notes:** per model the *p*-value and R^2^ value are shown. * = *p* < 0.05. **Abbreviations:** WAIS-IV, Wechsler Adult Intelligence Scale, fourth edition; VCI, Verbal Comprehension Index; PRI, Perceptual Reasoning Index; AWMI, Auditive Working Memory Index; PSI, Processing Speed Index; FSIQ, Full Scale Intelligence Quotient; FI, Fluid Intelligence; SMI, Short-term Memory; VIP, Visual Information Processing; WMS-IV, Wechsler Memory Scale fourth edition; AMI, Auditive Memory Index; VMI, Visual Memory Index; VWMI, Visual Working Memory Index; IMI, Immediate Memory Index; DMI, Delayed Memory Index.

#### 3.2.5. Non-Linear Effect of Age on Cognitive Functioning After Six Weeks Abstinence

When examining the potential non-linear relation between age and cognitive functioning across education levels, no significant models were identified for participants with a *low education level*.

Among participants with an *average education level*, the spline model indicated that age significantly predicted SMI (F(3, 57) = 4.08, *p* = 0.011, R^2^ = 0.18), pointing toward a non-linear pattern. Although none of the individual spline segments reached significance (excess40: B = −0.65, *p* = 0.267; excess50: B = 2.53, *p* = 0.061; excess60: B = −1.23, *p* = 0.504), the segment after age 50 (95% CI [−0.12–5.18]) showed a near significant positive trend.

Among participants with a *high education level*, the linear spline model indicated that age significantly predicted PRI, F(3, 27) = 3.00, *p* = 0.048, R^2^ = 0.25. While no significant contributions were found after the age of 40 and 50 (excess 40: B = 0.55, *p* = 0.569; excess50: B = 0.66, *p* = 0.734), a near significant trend for a decrease in the outcome was found after the age of 60 (excess 60: B = −3.67, *p* = 0.056, 95% CI [−7.45–0.11]).

#### 3.2.6. Effect of Age on Recovery of Cognitive Functioning

MoCA scores after two and six weeks of abstinence were available for 110 participants (see Table 2). When examining the potential relation between age and the degree of recovery, analysis yielded a statistically significant result (χ^2^(3) = 12.302, *p* = 0.006, Nagelkerke pseudo R^2^ = 0.115).

Compared to participants who showed no signs of improvement, each additional year of age was associated with the following:

- A near significant decrease in the odds of belonging to the improved group (*B* = −0.41, SE = 0.24, Wald χ^2^(1) = 3.04, *p* = 0.081, OR = 0.96, 95% CI [0.92, 1.01]), corresponding to approximately a 4% decrease in odds per year.
- A significant decrease in the odds of belonging to the group that deteriorated (*B* = −0.97, SE = 0.34, Wald χ^2^(1) = 8.21, *p* = 0.004, OR = 0.91, 95% CI [0.85, 0.97]), corresponding to approximately a 9% decrease in odds per year.
- A significant decrease in the odds of belonging to the group without any cognitive impairments (*B* = −0.47, SE = 0.19, Wald χ^2^(1) = 6.05, *p* = 0.014, OR = 0.96, 95% CI [0.92, 0.99]), corresponding to approximately a 5% decrease in odds per year.

## 4. Discussion

The present study investigated age-related deviations in cognitive functioning and cognitive recovery across cognitive domains commonly affected in patients with AUD. Participants were six weeks abstinent. A total of 40% of participants showed no improvement or even a decline in cognitive functioning, and substantial proportions with low (up to 71.4%) or average education (up to 34.9%) continued to perform below normative levels. Age-related deviations were identified for perceptual reasoning (PRI), verbal comprehension (VCI), and short-term memory (SMI). Specifically, a non-linear association between age and PRI was found in highly educated participants, while age-related improvements in VCI and SMI were observed in participants with high and average education levels, respectively. For SMI, non-linear effects indicated a specific change after age 50.

We investigated cognitive domains often affected in patients with AUD (working memory, auditive and visual memory, and visuospatial functioning). Only visuospatial functioning (reflected by PRI) and working memory (reflected by SMI) could be partly explained by age-related deviations, but not in domains reflecting auditive and visual memory. These null effects may partly reflect recovery associated with abstinence, as certain cognitive functions, such as processing speed, have been shown to recover within weeks of cessation [23]. In contrast, recovery of memory-related functions may require longer periods of abstinence [23,50]. Consistent with this, a substantial proportion of participants in the current sample continued to perform below normative levels on memory and other cognitive domains. However, as this is not explained by age-related deviation, other factors contribute to the observed impairments. Chronic and excessive alcohol use may accumulate several risk factors, such as (repeatedly experiencing) nutritional deficiencies, seizures, and neurotoxic effects [51], as well as increased risk for cerebrovascular accidents [52], all of which may negatively affect cognitive function. Alternatively, pre-existing cognitive and neurobiological characteristics, such as smaller gray and white matter volume, may also contribute to the lower cognitive functions observed [53,54,55].

The data in our study suggest that age-related influences may be limited to specific functions and do not follow a uniform or easily interpretable pattern. The deviation observed in PRI, for example, resembles patterns reported in healthy, non-AUD populations [56]. However, because these results are based on age-normed scores, they may reflect a negative deviation relative to age-adjusted expectations. One possible explanation is that increasing age increases sensitivity to the neurotoxic and cognitive effects of alcohol [57,58], potentially compromising the integrity of frontal and parietal brain regions that are critical for these functions [59,60]. Given the near-significant result and the low number of older participants (>60, N = 7), we must interpret this finding with restraint.

For VCI, the observed pattern aligns with findings in the general population, where VCI typically improves from age 20 to around age 60 [56]. In our sample, however, younger participants performed lower than older participants, possibly reflecting less accumulated crystallized verbal knowledge during chronic alcohol use. Alcohol-related cognitive impairment at a young age seems unlikely [61], as age of onset of chronic drinking did not affect the results in our study. It also cannot be excluded that performances within the normal range still resemble functioning below premorbid levels in patients with chronic and excessive alcohol use.

A similar, though more complex, pattern emerged for SMI. While a slight increase with age was observed, non-linear analyses also revealed better performance among participants aged 50 to 60. This contrasts with the age-related decline in SMI reported in healthy populations [62,63,64]. Given the limited variance explained by the model, this finding may represent a chance observation rather than a systematic pattern.

Analyses of cognitive recovery indicated that age influenced the likelihood of belonging to specific recovery categories. Compared to the group participants who did not improve, each additional year of age decreased the odds of having no cognitive impairments by 5% and was associated with a near-significant 4% decrease in the odds of showing recovery. These results may indicate increased vulnerability to the neurotoxic effects of alcohol and/or a slower recovery of cognitive functioning at older ages [21,57]. Finally, a 9% decrease in the odds of deterioration, compared to no improvement, was found. This finding is counterintuitive and might be affected by the small sample size of the older adults (>70 years) combined with relatively more highly educated individuals amongst these older patients. This finding needs to be replicated in a larger sample to be able to draw conclusions.

Overall, with respect to the overarching theme of premature aging, we found that older participants with predominantly severe AUD had a reduced chance of cognitive recovery and a reduced chance of having no cognitive problems at all and that perceptual reasoning after the age of 60 may be particularly susceptible to (prolonged) impairment during abstinence. Although it is plausible to expect that brain vulnerability—especially regarding alcohol use—increases with advancing age, this pattern is not as evident in our data. It should be taken into account that cognitive recovery was assessed using a screening instrument (the MoCA) and that our sample size was relatively small. Further research is therefore required to reach more definitive conclusions. Future studies might include a larger sample in which older adults with severe AUD are compared with education-matched younger adults with AUD, as well as with an older control group without AUD, across multiple cognitive domains and preferably assessed at two time points. No support was found for the accelerated aging hypothesis which suggests that age in patients with AUD is associated with systematically lower cognitive functioning compared to age-related peers.

This explorative study came with the following strengths. Firstly, we used data from a sample of typical treatment-seeking AUD patients admitted for clinical care. The characteristics (mean age of 43.9, 25% female, and 82% severe AUD) resemble those of other empirical studies in this field of research [65,66,67], increasing the external validity of our study. Secondly, we conducted the same standardized methodology for every patient, consisting of timely administration of assessments, a strict period of abstinence, verification of abstinence by means of urinalysis, and using well-established, widely used neuropsychological tests. The following limitations should be considered as they might have affected the validity of our findings. Firstly, we used age-normed scores to detect effects of age on cognitive functioning. This is a reasonable alternative, although a well-matched control group would have been preferable. While the instruments and the normed scores are well-accepted and widely used, participants with a higher level of education were more present when constructing the norm groups [68]. This may have lowered the sensitivity of the instruments to capture (subtle) differences in functioning among patients with lower education levels. In addition, the somewhat older participants in the high education group may also have affected the results, though the difference does not seem very big (about 8 years), and the distribution of age in the other education level groups is comparable. Given the need for stratification by education level in our study, future studies should aim to include controls matched for age, gender, and education level. Due to stratification, some of the analyses consisted of few observations, reducing power and limiting the detection of effects. The data on memory (WMS data) were especially limited, as was the size of the populations in the different recovery groups. The null results found may therefore be a result of low power (type II error) and should therefore be considered for replication in future research accounting for multiple testing to reduce type I error. Thirdly, our population consisted of only 2% of participants 70 years and older, while it is known that cognitive aging progresses after the age of 70 for several cognitive functions (reasoning, spatial visualization, memory, speed) [56,69]. Including older participants with AUD in empirical studies is therefore an important direction for future research, preferably using a multi-assessment design to capture recovery processes.

## 5. Conclusions

To conclude, our study predominantly found age-related effects on three cognitive indices (PRI, VCI and SMI) but not on ten other cognitive indices. Age-related effects in patients with predominantly severe AUD may be domain-specific, since only perceptual reasoning seems to be negatively affected after the age of 60 at six weeks of abstinence. Furthermore, we found that increasing age in this group of patients may reduce the odds of recovery and of not having any cognitive impairment at all. With regard to premature aging, our results do not support accelerated aging. Some of our findings may be consistent with the vulnerability hypothesis; however, due to study limitations we generally consider the results too uncertain to serve as evidence. Future studies should use control groups, include older patients with AUD, and account for age and education level to gain a more refined picture of cognitive aging during abstinence.

## Figures and Tables

**Table 1:** Demographic and drinking-related variables (n = 197).

Variable		N
Gender, n (%)		197
Men	145 (74%)	
Women	52 (26%)	
Age (years) [Mean (*SD*)]	43.9 (12.8)	196
19–29 years, n (%)	28 (14%)	
30–39 years, n (%)	52 (27%)	
40–419 years, n (%)	50 (26%)	
50–59 years, n (%)	42 (21%)	
60–69 years, n (%)	20 (10%)	
70–79 years, n (%)	4 (2%)	
Education level, n (%)		196
Low (Verhage 1–3)	39 (20%)	
Average (Verhage 4–5)	117 (59%)	
High (Verhage 6–7)	40 (21%)	
Severity of AUD n (%)		189
Light	8 (4%)	
Moderate	26 (14%)	
Severe	155 (82%)	
Years of daily alcohol use [Mean (*SD*)]	13.7 (11.2)	171
Age of onset of daily alcohol use [Mean (*SD*)]	28.3 (12.3)	171
Polydrugs use, n (%)	77 (42%)	183
Tobacco use disorder, n (%)	138 (70%)	182
Neurological injury, n (%)	25 (13%)	165

**Notes:** for the purpose of this study, the original seven categories of Verhage [32] were merged into three categories: Low (levels 1, 2 and 3), Average (levels 4 and 5), High (levels 6 and 7). **Abbreviations:** *SD*, Standard Deviation; AUD, alcohol use disorder.

**Table 2:** Overview mean age for four recovery categories (n = 110).

	Improved (19)	Not Improved (34)	Deteriorated (10)	No CI (47)
Age [Mean, (*SD*)]	43.53 (12.04)	49.88 (13.54)	35.70 (11.20)	42.66 (11.95)

**Notes:** Group assignment was based on a combination of two scores on a cognitive screening instrument administered in weeks 2 (T1) and 6 (T2) of clinical admission. Scores are either above or under cut-off. T1 score < cut-off and T2 score cut-off = Improved. T1 score < cut-off and T2 score < cut-off = Not Improved. T1 score cut-off and T2 score < cut-off = Deteriorated. **Abbreviations:** *SD*, Standard Deviation; No CI = no cognitive impairments.

**Figure A1:**
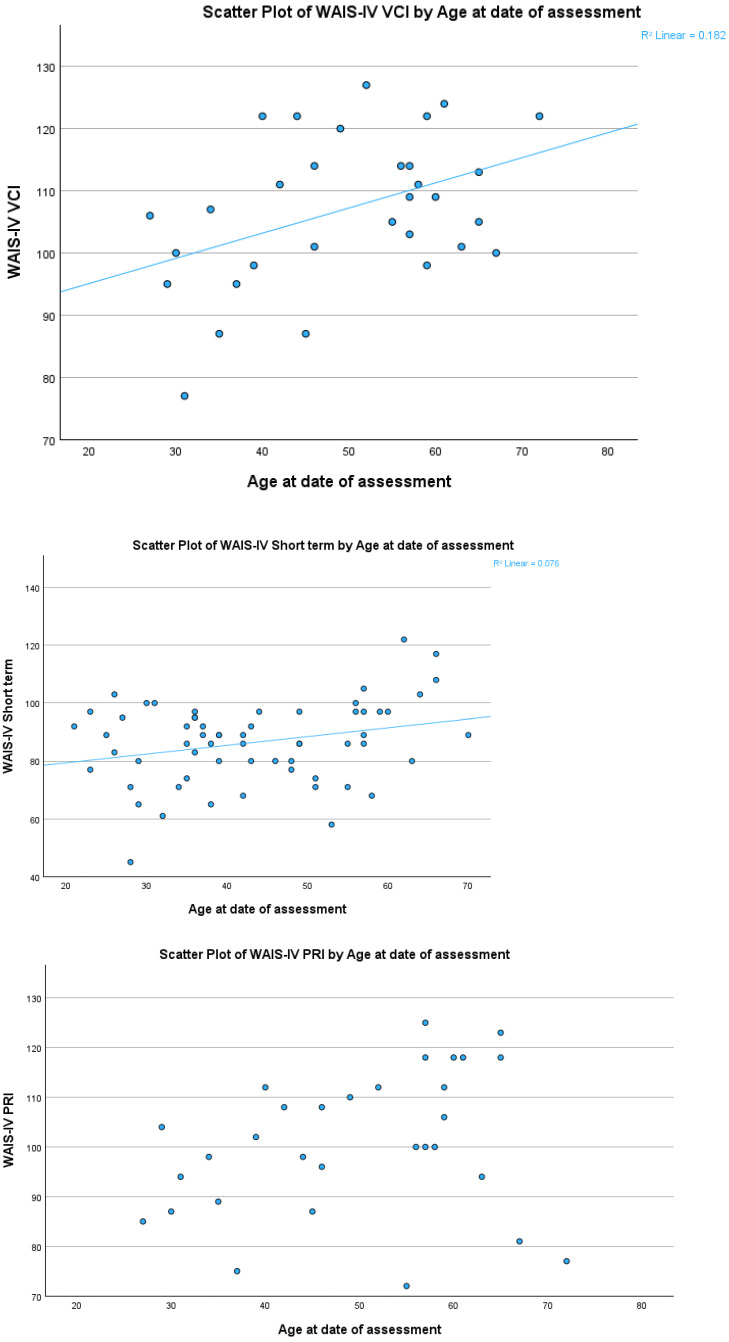
Scatterplots of cognitive tasks vs. age at the date of assessment.

## Data Availability

The data that support the findings of this study contain potentially identifying and/or sensitive patient information and hence are only available upon reasonable request to the scientific committee of Tactus Addiction Treatment (weco@tactus.nl). The provision of data will be considered by the Tactus Local Scientific Research Committee and the authors involved in this study. The sharing of patient data is subject to Dutch and European legal and ethical regulations.

## Appendix A

**Table A1:** Results: Descriptives of participants.

Cognitive Performance of Total Sample After Admission of Six Weeks						
	N	Min	Max	Mean	SD	<1.5 SD (%)
WAIS-IV						
VCI	148	60	129	90.46	15.443	16.9
PRI	147	60	125	86.68	14.967	27.9
AWMI	147	55	125	87.08	15.996	21.8
PSI	148	45	141	85.89	16.126	27.7
FSIQ	144	8	129	84.29	16.432	34.7
FI	97	54	127	83.82	16.029	37.1
SMI	96	45	125	86.77	16.503	24.0
VPI	96	56	127	86.45	13.800	27.1
WMS-IV						
AMI	93	0	145	32.30	30.380	24.7
VMI	93	0	119	34.24	29.899	20.4
VWMI	69	0	113	29.01	27.336	24.6
IMI	93	0	142	31.06	30.978	31.2
DMI	91	0	130	31.38	30.162	27.5

**Notes:** WAIS-IV normed scores are shown using T-scores (Mean = 100, SD = 15). WMS-IV normed scores are shown using percentiles. Scores < 1.5 Standard Deviation, ≈7% in a normal distribution, are scores ≤76 or ≤5th percentile. **Abbreviations:** WAIS-IV, Wechsler Adult Intelligence Scale, fourth edition, VCI, Verbal Comprehension Index, PRI, Perceptual Reasoning Index, AWMI, Auditive Working Memory Index, PSI, Processing Speed Index, FSIQ, Full Scale Intelligence Quotient, FI, Fluid Intelligence, SMI, Short-term Memory, VIP, Visual Information Processing, WMS-IV, Wechsler Memory Scale fourth edition, AMI, Auditive Memory Index, VMI, Visual Memory Index, VWMI, Visual Working Memory Index, IMI, Immediate Memory Index, DMI, Delayed Memory Index, SD, Standard Deviation.

**Table A2:** Confounders influencing the relationship between age and cognitive functioning.

Age Distribution per Education Level								
		Low (N = 39)			Average (N = 116)		High (N = 40)	
Mean		39.6			44.1		47.4	
Median		40.0			43.5		47.0	
Mode		42.0			42.0		57.0	
Std. Deviation		11.9			12.7		12.9	
Variance		140.9			162.1		165.8	
Range		51.0			54.0		48.0	
Minimum		19.0			19.0		24.0	
Maximum		70.0			73.0		72.0	
Percentiles	25	31.0			35.0		35.0	
	50	40.0			43.5		47.0	
	75	47.0			54.0		57.8	
**Effect of age of onset in the basic model stratified by education level**								
	**Average**				**High**			
Outcome	Age	AOO	R^2^	*p* Model	Age	AOO	R^2^	*p* Model
VCI	-	-	-	-	0.008 *	0.638	0.270	0.023 *
SMI	0.045 *	0.596	0.114	0.041 *	-	-	-	-
VWMI	0.100	0.867	0.078	0.230	0.064	0.643	0.279	0.166
**Effect of polysubstance use in the basic model stratified by education level**								
	**Average**				**High**			
Outcome	Age	PU	R^2^	*p* Model	Age	PU	R^2^	*p* Model
VCI					0.018 *	0.575	0.197	0.058
SMI	0.005 *	0.354	0.135	0.019 *				
VWMI	0.102	0.907	0.082	0.198	0.082	0.327	0.290	0.152

**Notes:** * = *p* < 0.05. **Abbreviations:** AOO, Age of Onset, VCI, Verbal Comprehension Index, SMI, Short-term Memory Index, VWMI, Visual Working Memory Index, PU, polysubstance use.
